# Understanding Arctic–Alpine Plants from Ecological and Evolutionary Perspectives

**DOI:** 10.3390/plants13111560

**Published:** 2024-06-05

**Authors:** Gregor Kozlowski

**Affiliations:** 1 Department of Biology and Botanical Garden, University of Fribourg, Chemin du Musée 10, CH-1700 Fribourg, Switzerland; gregor.kozlowski@unifr.ch; 2 Natural History Museum Fribourg, Chemin du Musée 6, CH-1700 Fribourg, Switzerland; 3 Eastern China Conservation Centre for Wild Endangered Plant Resources, Shanghai Chenshan Botanical Garden, 3888 Chenhua Road, Songjiang, Shanghai 202602, China

Conditions in arctic and alpine ecosystems impose great challenges on the plants and other organisms that live there [1]. Despite this, thousands of plant species worldwide survive or even prosper under extreme climatic, edaphic, and ecological conditions in the High North (or South) and in the high mountains [2]. Arctic and alpine plants continue to surprise researchers with their ingenious strategies and adaptations [3]. Today, global warming, the ever-increasing demand for resources, and the expansion of tourism are growing threats to arctic and alpine plant life, even in the most remote regions of the world. This applies not only to glacial relics and endemics in isolated mountain refugia but also to tundra areas that were intact until recently and are now under increasing pressure from man-made global changes [4,5]. In such a context, this Special Issue of *Plants*, entitled *Arctic and Alpine Plants: Ecology, Adaptations and Conservation Biology*, has been launched to improve our understanding of this unique group of plants from ecological, biogeographical, and evolutionary perspectives.

A publication by Körner [6] provides an excellent opportunity to investigate the complexity and specificity of high mountain plants and their communities, which are often found at the edge of their fundamental niches. This review paper presents no less than 12 concepts in alpine plant ecology, starting with life forms and aspects of the physical environment, such as topography, moving through physiology and reproductive biology, and finishing with global change drivers and their influence on plants growing in the alpine ecological zone. Despite the enormous complexity of such factors, Körner [6] states in his concluding comments that “nothing make sense in alpine plant biology unless one accounts for micro-climate”. This is further developed upon in concept 11 of his review, entitled “To be or not to be—the edge of the fundamental niche”. The author postulates that any projections of future species distributions, especially in the alpine and arctic context, need to rest on a very fine-grained representation of the landscape and stresses the essential role of microtopography in any modeling study.

The second review paper, by Hörandl [7], presents so-called geographical parthenogenesis and explores its importance for alpine and arctic plants. Geographical parthenogenesis describes the phenomenon of asexual organisms usually having larger distribution areas than their sexually reproducing relatives and colonizing more frequently at higher latitudes and in previously glaciated or otherwise devastated areas. However, the causal factors behind this phenomenon are still unclear. Polyploidy, epigenetic flexibility, and phenotypic plasticity are often seen as important factors fostering geographical parthenogenesis patterns. Additional large-scale studies are needed that include information on reproduction and that integrate ecological, experimental, and molecular research involving many genera and large-scale screening from large geographical areas. High arctic regions are of great importance since they are not easily accessible and, thus, are not well explored.

Polyploidy is an important phenomenon in understanding the biogeography and taxonomy of disjunct arctic–alpine species complexes. Wagner et al. [8] provided detailed insights into taxonomically challenging hexaploid alpine shrub willows (*Salix* sp., *Salicaceae*). In this study, RAD sequencing data, infrared spectroscopy, and morphometric data were used to analyze the phylogenetic relationships of the species from the *Phylicifoliae* and *Nigricantes* sections in a framework of 45 Eurasian *Salix* species. The results reveal, among other insights, that both sections are polyphyletic and need to be redefined. Additionally, *S. bicolor* should be included in *S. phyllicifolia* s.l., and the alpine endemic *S. hegetschweileri* is a distinct lineage showing close relationships to the *Nigricantes* clade.

The importance of large-scale geographical sampling when studying disjunct and polyploid arctic–alpine plant complexes was confirmed by two studies performed by Kozlowski et al. [9,10]. These two studies explored the genome sizes and ploidy levels of the *Arenaria ciliata* species complex (*Caryophyllaceae*). Their papers enabled the first synthesis of the ploidy level variability within this group, concluding that three taxa are predominantly tetraploid (2n = 4x = 40): the arctic *A. pseudofrigida* and the two alpine taxa *A. multicaulis* and *A. ciliata* subsp. *ciliata*. Higher ploidy levels were detected in *A. norvegica* (2n = 8x = 80) and *A. gothica* (2n = 10x = 100), and the highest detected ploidy level was for the narrow endemic of the Western Alps *A. ciliata* subsp. *bernensis* (2n = 20x = 200). These results confirm that there are no diploid taxa within the *A. ciliata* species complex and that this group is an example of a so-called *mature polyploid complex,* a common phenomenon among arctic–alpine taxa. The *A. ciliata* species group is, thus, an example of an arctic–alpine species complex characterized by reticulate evolution, polyploidization, and hybridization, most likely associated with rapid latitudinal and altitudinal migration from the Pleistocene through to the Holocene.

Due to their adaptations to cold environments, arctic and alpine plants are very sensitive to climate change. Two papers published in this Special Issue explore the influence of changing climate conditions on the past and future distributions of plants growing at high latitudes and altitudes. Both studies use traditional species distribution models (SDMs) based on data extracted from the Global Biodiversity Information Facility (GBIF) and, thus, do not use fine-grained representations of the landscape, as proposed by Körner [6]. Walas et al. [11] assessed and compared the current and future potential niche areas of *Kalmia procumbens* (*Ericaceae*) in the Pyrenees and Carpathians. Both mountain ranges represent the southernmost localities of this circumpolar, arctic–alpine species in Europe. The results demonstrated, as expected, that the species covered a larger distribution area during the Last Glacial Maximum; due to recent, accelerated climate warming, a reduction in the potential distribution area is expected by 2100. However, the reduction will be substantial in the Carpathians, where only a few South Carpathian populations would persist, and would be rather moderate in the Pyrenees.

Zhang and Wang [12] presented another study using SDMs, aiming to predict the potential distribution of endangered *Meconopsis punicea* (*Papaveraceae*), which is endemic to China and grows in high-altitude grasslands and shrublands between 2800 and 4300 m a.s.l. Four types of species distribution models were applied: generalized linear model (GLM), generalized boosted model (GBM), random forests (RF) and flexible discriminants analysis (FDA). Under future climate change, the potential distribution of *M. punicea* will expand from southeastern to northwestern China. However, there were significant differences in the distributions predicted by the different SDMs. Therefore, the authors propose the use of consistent results from different SDMs as the basis for developing conservation strategies.

In addition to the frequently covered effects of climate warming, many other human-made changes have important impacts on the survival of arctic and alpine plants. Chardon et al. [13] explored the response of arctic shrubs and graminoids to human trampling, an integral component of global change; however, a comprehensive understanding of the effects of trampling on alpine and arctic ecosystems is lacking. The authors surveyed trail-side (disturbed) and off-trail (undisturbed) transects along altitudinal gradients in the Garibaldi Provincial Park in Canada. The main focus was placed on dominant shrubs (*Phyllodoce empetriformis*, *Cassiope mertensiana*, and *Vaccinium ovalifolium*, all three of which are *Ericaceae*) and on graminoids (*Carex* species, *Cyperaceae*). The study demonstrated that trampling reduces plant cover in all species and that growth traits are more sensitive than reproductive traits. However, the disturbance response is species-specific. For example, a reduction in size was observed only for *P. empetriformis* and *V. ovalifolium*, whereas in *C. mertensiana,* human trampling mainly reduced reproductive output. Interestingly, in *Carex* spp., height, but not diameter, was sensitive to trampling, indicating the need to measure the traits of multiple species to better understand the effects of disturbance on arctic and alpine plants. The results of this study are of great conservation importance, highlighting that specific management approaches may need to be tailored toward the protection of particular species.

Another aspect linked to land-use changes and climate warming is the spread of shrubs in arctic and alpine environments. Oberhuber et al. [14] focused on the most rapidly expanding shrub species in the European Alps, the green alder (*Alnus alnobetula*, *Betulaceae*). This study, using dendrochronological methods, was carried out in alder populations spread above 2100 m a.s.l. within the alpine treeline ecotone on Mt. Patscherkofer (Tyrol, Austria). The main problem in such high-altitude individuals is their asymmetric radial growth and anomalous growth ring patterns, which make representative measurements very challenging. Consequently, it is very difficult to differentiate between the influences of climate and microsite conditions on radial growth. In topographically heterogeneous environments, site-specific variability in the climate–growth relationships of *A. alnobetula* must be considered. Therefore, the authors provide the following sampling recommendations when undertaking investigations on site-specific variability: measuring one radius per shoot, three shoots per individual, and a minimum of ten individuals within the same stand, which results in ca. ten radii per study plot. However, for the determination of absolute growth rates and growth trends with respect to recent climate warming, the following recommendations are made: measuring 4 radii per shoot, ≥5 shoots per individual, and ≥10 individuals within one stand (total ca. 200 radii per study plot).

Another important phenomenon in alpine ecosystems is the decrease in the diversity of life forms with altitude. López et al. [15] investigated the changes of trees to shrubs and other life forms along an elevational gradient. The authors used *Polylepis tarapacana* (*Rosaceae*), a small, cold-tolerant, evergreen tree species growing in the Andes in the high-elevation Altiplano as a model taxon. After Tibet, the Altiplano Plateau, which lies between Bolivia, Peru, and Chile, is the second most extensive high plateau on Earth. In northwestern Argentina, where the study was carried out, *P. tarapacana* forms monospecific stands in a treeline ecotone. The authors propose a new classification of life forms for this species: arborescent, shrub, dwarf shrub, and *brousse tigrée* (tiger bush). The justification for this new classification lies not only in the marked biometric characteristics but also in the different influences of topographic, climatic, and human-use factors on their frequency. Therefore, it is important to consider these different life forms of *P. tarapacana* when conducting studies and developing management plans, since each life form occupies a particular environmental situation. The authors concluded that the conservation of *P. tarapacana* forests without differentiating between their life forms is achieved through the loss of part of their ecological niche.

In summarizing the subject matter presented in this Special Issue of *Plants*, entitled *Arctic and Alpine Plants: Ecology, Adaptations and Conservation Biology*, it is evident that, despite the long tradition of alpine and arctic research, there is still much to be discovered. The future of these highly specialized organisms is uncertain. All the authors of this Special Issue agree that much more intensive field work and field research should be carried out, as well as more experimental work. Arctic–alpine plants and vegetation can serve as a barometer of global change. Therefore, research on this group of plants is highly valuable. 
